# Quality Improvement for Outpatient COVID-19 Infection Control

**DOI:** 10.7759/cureus.16373

**Published:** 2021-07-13

**Authors:** Jeremy Clay, Kelly Morton, Daniel Franz, Ecler Jaqua, Van Nguyen

**Affiliations:** 1 Family Medicine, Loma Linda University Medical Center, Loma Linda, USA; 2 Psychology, Loma Linda University Medical Center, Loma Linda, USA

**Keywords:** covid-19, quality improvement research, disease control, control, infection control guidelines, infectious control

## Abstract

Background

To stop the spread of COVID-19 in outpatient primary care clinics, infection control strategies were needed including social distancing and masking in Fall 2020. Studies show a significant decrease in COVID-19 transmission when healthcare professionals comply with preventive measures. We tested whether an educational video would improve compliance to infection control behaviors quickly.

Objective

To improve COVID-19 infection control compliance in clinical staff at an outpatient federally qualified health center (FQHC) family medicine residency clinic with quality improvement (QI) tools.

Methods

On-line surveys assessed medical assistants’ (MAs), residents’, and attending physicians’ before and after an educational video intervention to assess knowledge of and compliance with social distancing and masking guidelines. Independent observed compliance assessments before and after the educational video were used to confirm the self-reported compliance.

Results

The pre- and post-intervention surveys were completed by 49% (37/76) and 62% (47/76) of participants, respectively. Self-reported knowledge and compliance showed no significant change over time. Observed compliance, however, from pre (n = 667) to post (n = 1132) intervention improved for both masking (p < 0.001) and social distancing (p < 0.001).

Conclusion

An educational video regarding COVID-19 infection control was effective in improving compliance in an outpatient clinic in an underserved, urban setting. While building these new behavioral habits, however, self-report may not be as accurate as observational assessments. Since this intervention was implemented prior to the COVID-19 fall surge and introduction of mass vaccinations, the educational intervention may have improved behavioral compliance with COVID-19 protocols later in the pandemic.

## Introduction

With the declaration of the COVID-19 pandemic in March 2020, a rash of infection control measures was initiated, focusing on the inpatient setting. For an outpatient clinic, however, recommendations for physical distancing and face masks required overnight changes to maximize the safety of clinicians, staff, and patients so that patient access to in-person care could be maintained to prevent unnecessary ED visits.

The setting of this project was a family medicine residency clinic within a large, urban federally qualified health center (FQHC) where resident physicians provide most of the direct patient care with supervision from attending physicians, all working together with patient service representatives (PSRs), medical assistants (MAs), and licensed vocational nurses (LVNs). It is a diverse, medically underserved community, predominately Hispanic/Latino at 53% and non-English speaking at 21%. Moreover, approximately 31% of the FQHC patient population does not have internet access for telehealth; it was critical to continue to offer in-person visits and enhance infection control strategies [1].

In response to the COVID-19 pandemic, the FQHC implemented a multi-pronged plan to reduce the risk of COVID-19 transmission: (1) all entering the facility had temperature and symptom screenings, (2) based on CDC guidance, all must wear a mask, (3) standard of care handwashing or hand sanitizer use between any contact with patients or adjusting masks (e.g., gel in, gel out, gel often) was encouraged, (4) social distancing of six feet was maintained if unmasked and a face shield was used if unmasking was necessary for a patient procedure, and (5) telehealth visits were rapidly integrated into the clinic workflows.

This quality improvement (QI) project focused on staff and clinician masking and social distancing for several reasons. First, a Cochrane review found that one-time symptom and temperature screening could potentially miss between 40 to 100% of infected individuals who may be asymptomatic but infectious [2]. Second, a prospective cohort study found that healthcare workers have an adjusted hazard ratio of 3.4 compared to the general population with personal protective equipment (PPE) reducing but not mitigating the risk of viral transmission [3]. Third, a meta-analysis suggests that social distancing and masking reduce the risk of COVID-19 spread by 82% (aOR = 0.18) and 85% (aOR = 0.15), respectively. Finally, statistical modeling suggests that 80-90% compliance for masking in the general population is required to reduce the reproductive number (R0) of the virus to less than 1, the threshold to stop the spread of the pandemic [4]. With this information, infection control needed to be addressed swiftly and effectively through social distancing and masking to mitigate the transmission of and exposure to COVID-19 while protecting both the healthcare team and potential patients [5].

In informal focus groups, the healthcare team reported inconvenience and mask discomfort as the main reasons for poor infection control compliance. Several members of the team questioned the efficacy of social distancing in infection control. To address these concerns and potentially invoke change in infection control perceptions and rates of compliance with social distancing and masking, we decided that knowledge, perceived disease risk, efficacy of preventive behaviors, disease treatment efficacy, and social norms would all influence staff/physician infection control behaviors [6-9]. To address these factors, we created an educational video that could be easily shared to educate staff/physicians on the efficacy of the infection control behaviors, the risk and outcomes of the disease, and the correct infection control behaviors. We hypothesized that perceived and observed infection control behaviors of masking, social distancing, and hand hygiene would improve from before to after the educational video within eight weeks.

## Materials and methods

Participants 

The FQHC is comprised of 76 team members that include MAs, LVNs, and residents and attending physicians. All team members were included in the observational phase of the project. A total of 37 (48.7%) and 47 (61.8%) team members participated in a 13-item pretest survey and 15-item post-test survey, respectively. Team member responses were further sorted into staff (MAs and LVNs) and physicians (residents and attendings).

Measures

Surveys measured self-reported compliance to social distancing and masking, assessed knowledge of compliance behaviors, and gauged the viewing of the educational video. Using a 5-point scale, 1 = 0-20%, 2 = 21-40%, 3 = 41-60%, 4 = 61-80%, 5 = 81-100%, participants answered two questions about compliance to social distancing and masking. Knowledge about COVID-19 transmission was assessed with three questions in a “check all the apply” format, scores ranged from 0 to 11 points. The post-test survey included two questions regarding the educational video and signage for COVID-19 precautions.

Observed compliance with masking and social distancing was measured by team members consisting of physicians and doctoral students who are normally in the clinic and thus not disruptive to regular clinic flow. Observers were trained to record observed compliance behaviors in the staff break room, general pods, and hallways at random times in the clinic using a tally method. The number of team members, the members’ role, and compliance with both social distancing and masking were measured in each of the aforementioned areas. There were 60 observation sessions before the educational video was introduced and 77 observation sessions after.

Procedure

**Figure 1:**
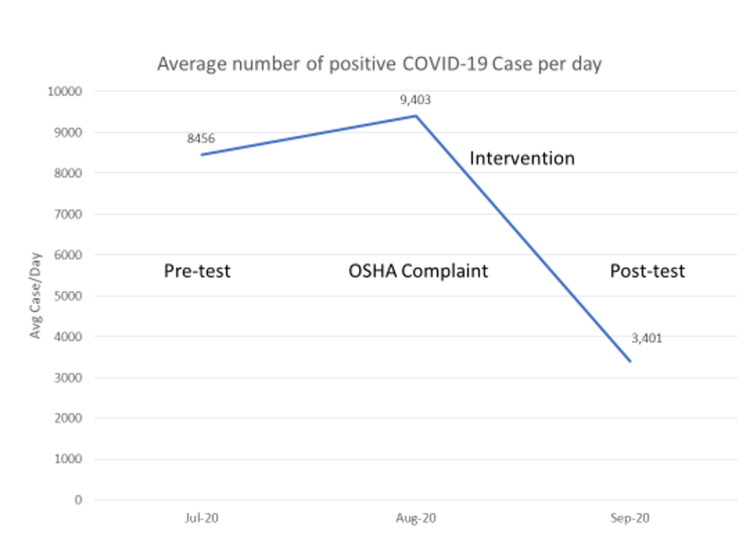
Timeline of measures, intervention, and relevant COVID-19 rates. NOTE: Case count is a monthly average for California.

An educational video about COVID-19 infection control behaviors was created to enhance knowledge and improve compliance with CDC recommendations of social distancing and masking. Methods of infection transmission, proper handwashing techniques, and the use of face shields were also included in the video. The training was based on clinic policies and guidelines provided by the CDC and California Department of Public Health in fall 2020. In addition, screen savers and posters were placed throughout the clinic as visual cues to encourage compliance with infection control behaviors. Lastly, following an Occupational Safety and Health Administration (OSHA) complaint regarding poor masking compliance, a clinic policy mandating immediate removal from the clinic if infection control behaviors were implemented in August 2020. Relevant COVID-19 statistics related to the time of intervention are provided (Figure 1).

## Results

Data were analyzed using SPSS Version 27 with statistical significance set at p < 0.05. A paired-sample t-test was used to separately assess changes in knowledge and self-reported compliance scores before and after the intervention. A chi-square test of independence was used to test for associations between each observed compliance behavior before and after the intervention. Logistic regression was used to assess ORs for masking and social distancing before and after the intervention. There were no outliers or violations of assumptions reported.

The pretest survey response rate was 44% (8/18) for staff and 50% (29/58) for physicians; the overall pretest response rate was 49% (37/76). The post-test survey response rate was 61% (11/18) for staff and 62% (36/58) for physicians; the overall post-test response rate was 62% (47/76). 

Staff scored 93% on the pretest and 89% on the post-test knowledge test; 76% reported viewing the educational video and 79% saw posters or screen savers. Physicians scored 94% on the pretest and 92% on the post-test knowledge test; 72% reported viewing the educational video and 75% saw posters or screen savers. There was no significant difference, p > 0.05, in knowledge scores after intervention for either group. 

Table 1 shows the rates of self-reported masking, social distancing, and hand hygiene by group on the 1-to-5-point rating scale. Staff reported no change in infection control behaviors from the pre- to the post-test period. Physicians, however, showed a significant increase in self-reported compliance to social distancing after the intervention period, p < 0.05. 

Observational data were collected pre (N = 667 observations) and post (N = 1132 observations) intervention. Staff and physician compliance increased significantly from the pre- to the post-intervention period in masking and social distancing (Table 2).

**Table 1:** Self-reported infection control compliance by group. NOTE: 1 = 0-20%, 2 = 21-40%, 3 = 41-60%, 4 = 61-80%, 5 = 81-100%. *p < 0.05.

Prevention Behavior	Staff (n = 5)		Physicians (n = 26)	
	Pre-test	Post-test	Pre-test	Post-test
Mask Use	5	5	4.8	4.8
Social Distancing	4.6	4.0	4.5	4.7*

**Table 2:** Observed infection control compliance by group. NOTE: ***p < 0.001.

Prevention Behavior	Staff			Physicians	
		Pre-test (%)	Post-test	Pre-test	Post-test
Mask Use		84.7	94.2***	96.1	99***
Social Distancing		85.24	93.8***	96.1	99***

With regard to social distancing, across team members, there was a significant increase from 88.2% to 95.7% in observed compliance after the intervention (χ2 (1, N = 1799) = 34.8, p < 0.001). All observed groups were 2.9 times more likely to follow social distancing guidelines after the intervention (OR = 2.9, 95% CI [2.0, 4.2], p < 0.001). By clinic role, MAs and LVNs were 2.9 times more likely to follow social distancing guidelines after the intervention (OR = 2.9, 95% CI [1.9, 4.3], p < 0.001). Physicians were 4.4 times more likely to follow social distancing guidelines after the intervention (OR = 4.4, 95% CI [1.3, 14.4], p < 0.05. 

And, with regard to masking infection control behavior, across all team members, there was a significant increase from 89.2% to 95.9% in observed compliance after the intervention (χ2 (1, N = 1799) = 30, p < 0.001). All observed groups were 2.8 times more likely to follow masking guidelines after the intervention (OR = 2.8, 95% CI [1.9, 4.1], p < 0.001). By clinic role, MAs and LVNs were 2.7 times more likely to follow masking guidelines after the intervention (OR = 2.7, 95% CI [1.8, 4.1], p < 0.001). Physicians were 4.4 times more likely to follow masking guidelines after the intervention (OR = 4.4, 95% [1.3, 14.4], p < 0.05).

## Discussion

The COVID-19 pandemic brought a need for improved infection control in both the inpatient and outpatient settings at a magnitude not seen in recent times. Due to the urgency of this need, multi-component interventions were needed to not only mitigate the potential transmission of COVID-19 within the clinic but also protect those working within it. This QI project showed a significant improvement in infection compliance behaviors after an educational video and visual cues were distributed and policy changes were invoked. The efficacy of these interventions was observed in a family medicine residency clinic that serves the underserved; no known cases of COVID-19 originated in our clinic during this project.

Although the QI project occurred from summer to fall 2020, and the effective control measures against COVID-19 have since changed, we are confident that education lies at the heart of any successful intervention. Education of all team members as to the importance of infection control behaviors improves compliance with such mandates. Moreover, monitoring of these behaviors helped team members become more aware of how frequently a lapse from infection control recommendations occurred. Self-reported infection compliance did not perfectly align with observed compliance behavior improvements in social distancing or masking for the staff. Physician self-reports and observed behaviors were more similar before and after the intervention, with noted improvement in infection control behaviors. Although improvements in social distancing and masking were noted among all team members, it must be acknowledged that the improved rates may have also been a reflection of widespread media coverage and community pressure and regulations. The educational video, signage, and clinic policy changes were not the only sources of information regarding infection control behaviors. 

This QI project, although with several statistically significant interventions, has several limitations including real-time changes in infection control and prevention guidance, clinic policy changes at the level of the institution, and heavy media coverage regarding infection control behaviors. These factors may have contributed to the “decrease” in infection control behavior knowledge noted in the post-test surveys. Moreover, the scale on the self-report tool had a ceiling effect as the highest point on the scale crossed the actual levels of change in behavioral compliance. Therefore, the self-report data should be viewed with caution and should be replicated. Modification to the observation protocols, such as following a finite number of team members at any given time, including patient care areas, may be considered to improve the quality of observational data.

## Conclusions

Although improved COVID-19 infectivity rates are noted and vaccinations have been introduced, infection control and prevention behaviors remain vitally important to this day. As more information develops and the COVID-19 pandemic evolves, the behaviors of each team member will likely need to be adjusted according to new CDC recommendations and mandates. As such, this QI project demonstrates that surveys and observational tools can be effective and low-cost strategies to monitor and provide feedback to the healthcare team. Additional studies that examine the effectiveness of these interventions to reduce transmission of COVID-19 in different clinical settings may have applications to other areas of healthcare and in the community at large.
